# Queen's Jubilee Hospital

**Published:** 1894-08-25

**Authors:** 


### Editor's Letter-Box.

[Our correspondents are reminded that proxility is a great bar to publication, and that brevity of style and conciseness of statement greatly facilitate early insertion.]

### QUEEN'S JUBILEE HOSPITAL.

The Secretary writes: I notice in your issue of August 4th that the name of this hospital is marked "nil" in the list of the Hospital Sunday Fund awards. Permit me to mention that we received an award of £43 2s. 6d.

